# Jatrorrhizine Alleviates DSS-Induced Ulcerative Colitis by Regulating the Intestinal Barrier Function and Inhibiting TLR4/MyD88/NF-*κ*B Signaling Pathway

**DOI:** 10.1155/2022/3498310

**Published:** 2022-09-19

**Authors:** Shengqi Niu, Manyi Jing, Jianxia Wen, Shizhang Wei, Haotian Li, Xing Li, Xiao Ma, Yanling Zhao

**Affiliations:** ^1^Chinese PLA Medical School, Beijing, China; ^2^Department of Pharmacy, Chinese PLA General Hospital, Beijing, China; ^3^School of Food and Bioengineering, Xihua University, Chengdu, China; ^4^College of Pharmacy, Chengdu University of Traditional Chinese Medicine, Chengdu, China

## Abstract

**Background:**

Ulcerative colitis (UC), a kind of autoimmune disease with unknown etiology, has been troubling human physical and mental health. Jatrorrhizine (Jat) is a natural isoquinoline alkaloid isolated from *Coptis Chinensis*, which has been proved to have antibacterial, anti-inflammatory, and antitumor effects.

**Purpose:**

The purpose is to explore the therapeutic effect of Jat on DSS-induced UC and the mechanism of action. *Study Design.* The UC mice model was induced by 3% DSS in drinking water. The mice were orally administered with Jat (40, 80, 160 mg/kg) for 10 days.

**Methods:**

The changes in body weight, colon length, spleen wet weight index, disease activity index (DAI), colonic histopathology, and inflammatory factors of serum and colon tissue were analyzed to evaluate the severity of colitis mice. The colon mucus secretion capacity was analyzed by Alcian blue periodic acid Schiff (AB-PAS) staining. Furthermore, protein expressions such as TLR4, MyD88, p–NF–*κ*B-p65, NF-*κ*B-p65, COX-2, ZO-1, and Occludin were detected to elucidate the molecular mechanism of Jat on DSS-induced colitis model.

**Results:**

The results showed that Jat could significantly alleviate the symptoms, colon shortening, spleen index, and histological damage and restore the body weight in DSS-induced colitis mice. Jat also suppressed the levels of inflammatory cytokines and upregulated the levels of anti-inflammatory cytokines. In addition, Jat repaired the intestinal barrier function by upregulating the level of colonic tight junction (TJ) proteins and enhancing the secretion of mucin produced by goblet cells. Furthermore, Jat could significantly suppress the expression of TLR4, MyD88, p–NF–*κ*B-p65/NF-*κ*B-p65, and COX-2 in colon tissue.

**Conclusion:**

The results suggested that Jat plays a protective role in DSS-induced colitis by regulating the intestinal barrier function and inhibiting the TLR4/MyD88/NF-*κ*B signaling pathway. This study, for the first time, demonstrates the therapeutic and protective effects of Jat on UC.

## 1. Introduction

Ulcerative colitis (UC) is an inflammatory bowel disease affecting the colonic mucosa and submucosa, which has the characteristics of a long course, difficulty to cure, relapsing inflammation of the gastrointestinal, and the increasing risk of colon cancer [1]. The underlying causes of UC are unclear and complicated, and the exact pathogenesis of UC is still limitedly understood. The symptoms of UC are clinically characterized by abdominal pain, rectal bleeding, and diarrhea [2]. Drugs like salazosulfa pyridine, mesalamine, glucocorticoids, and biologic agents are the preferred treatment for UC, but none of them have achieved satisfactory effects for the majority of UC patients, and high price restrict their clinical application [3, 4]. Due to the high incidence of UC in developing countries and the high prevalence of UC in developed countries in recent years, this disease has evolved into a global public health concern [5, 6]. Thus, seeking out alternative or supplementary therapy, especially from natural drugs, is urgent for patients with UC.

The dextran sulfate sodium (DSS) model was first reported by Okayasu and other scientists [7]. Oral administration of 40–50 kDa DSS-containing water to mice could lead to clinical symptoms and intestinal epithelial morphology injury, which most closely resembles human UC [8]. Hence, mouse colitis induced by DSS has been widely used as an experimental model in UC research [9, 10]. Although the exact pathogenesis of UC is still poorly understood, increasing evidence indicated that the disruption of intestinal barrier function might contribute to the development of UC [5, 11, 12]. The intestinal mucosal barrier includes mucin proteins largely produced by goblet cells, intestinal epithelial cells (IECs), and immune cells in the lamina propria [13]. Tight junction (TJ) proteins consisting of ZO-1 and Occludin, which are located at the interface between epithelial cells, prevent pathogens and detrimental antigens from entering the body through the intestinal barrier [5, 14, 15]. In addition, the imbalance of inflammatory cytokines plays a vital role in UC patients and TLR4/MyD88/NF-*κ*B signaling pathway is critical in the development of UC [16–19]. Moreover, NF-*κ*B also plays an important part in the disruption of epithelial barrier function and interaction between mucosal immune response and gut microbiota [20]. Therefore, maintaining the integrity of the intestinal mucosal barrier is of great significance in the treatment of UC and the TLR4/MyD88/NF-*κ*B signaling pathway may be a potential therapeutic target for UC.

Zuojin Pill (ZJP), composed of *Coptis Chinensis* and *Fructus Evodiae* in a ratio of 6 : 1(g/g), as a classic prescription of Traditional Chinese Medicine (TCM), has been used to treat gastrointestinal diseases effectively for hundreds of years and also exhibited beneficial therapeutic effects on experimental colitis [21]. Jatrorrhizine (Jat) is an isoquinoline alkaloid, which is the main bioactive compound of Chinese herbs, such as *Coptis Chinensis*, and *Phellodendron Chinense Schneid* [22, 23]. In addition, Jat is a natural protoalkaloid that has a matrix structure with berberine, another alkaloid found in *Coptis Chinensis*. However, evidence has shown that Jat is a safe compound with lower cytotoxicity than berberine [24, 25]. Furthermore, a growing number of publications have demonstrated that Jat has a variety of pharmacological properties, including neuroprotective effects [26], antidiabetic effects [27], antirheumatoid arthritis effects [28], antimicrobial effects [29], anti-inflammatory effects [24, 30–32], antitumor effects [33, 34], and gut microbiota balances [35]. Despite the fact that Jat has many of the above biological activities, the effect of Jat on UC is unknown. In the present study, we evaluated the anti-inflammatory activity and the protective effect on the integrity of intestinal barrier integrity of Jat against DSS-induced colitis in C57BL/6 mice. Moreover, we also explored the possible underlying molecular mechanisms in this experiment. The findings may provide beneficial evidence for Jat as a curative drug for UC.

## 2. Materials and Methods

### 2.1. Ethics Statement

All animal procedures are conducted in strict accordance with the recommendations of the Guide for the Care and Use of Laboratory. All breeding and animal treatments complied with the Animal Ethical and Experimental Committee of the Fifth Medical Center of PLA General Hospital (Approval ID : IACUC-2019-004).

### 2.2. Reagents and Chemicals

DSS (36–50 kDa) was purchased from MP Biomedicals (Canada). Phospho–NF–*κ*B p65 Rabbit mAb (cat. no. 3033) and NF-*κ*B p65 Rabbit mAb (cat. no. 8242) were obtained from Cell Signaling Technology, Inc. TLR4 (19811-1-AP), zonula occludens-1 (ZO-1) (21773-1-AP), Occludin (27260-1-AP), and GAPDH (10494-1-AP) were obtained from Proteintech (USA). MyD88 (AF5195) was purchased from Affinity Biosciences (USA). COX-2 (bs-0732R) was obtained from BIOSS. Jat Standard (purity ≥98%, Cat. No. 6681-15-8) was purchased from Chroma Biotechnology Co. Ltd. (Chengdu, China). Mesalazine (5-ASA) was purchased from Losan Pharma GmbH (lot. no. L20235A). Jat and 5-ASA were dissolved in water to make an oral suspension. IL-1*β*, TNF-*α*, MPO, IL-10, and TGF-*β* enzyme-linked immunosorbent assay (ELISA) kits were purchased from Shanghai Enzyme-Linked Biotechnology (Shanghai, China) Co., Ltd.

### 2.3. Experimental Animals and Establishment of the DSS-Induced Colitis Model

Male C57BL/6 mice (8 weeks, 18 g–22 g) were purchased from Spiffy (Beijing) Biotechnology Co. Ltd. With reference to previous relevant studies, C57BL/6 mice were given 3% DSS in sterile water to establish the UC animal model. [8, 36, 37]. According to the experimental results of our preliminary experiment, 40, 80, and 160 mg/kg dosages of Jat were selected as drug discovery dose groups in this experiment. All animals were first adaptively fed in the SPF conditions (temperature: 25 ± 0.5°C, humidity: 55 ± 5%, 12 h: 12 h light dark cycle) for 7 days with adequate food and sterile water. After 1 week of acclimation, the mice were randomly separated into six groups (*n* = 10): control group, DSS group, Jat (40, 80, 160 mg/kg) + DSS groups, and 5-ASA (0.52 g/kg) + DSS group [38]. In brief, with the exception of the control group, mice in each group were given 3% DSS in drinking sterile water for 7 days followed by given normal drinking sterile water for 3 days. Jat and 5-ASA were taken orally once a day for 10 days at the same time as the model was started. The mice were euthanized on the last day.

### 2.4. Disease Activity Index (DAI)

The body weight, stool consistency, and occult bleeding of all mice in each group were observed daily, and the rate of body weight change and DAI score were calculated. The weight change refers to the comparison of the weight of the mice on the day of the test with the weight on day 0. DAI determined by combining rectal bleeding, stool consistency, and body weight loss scores, and the evaluation was based on the scoring system shown in Table 1 as previously described [39].

### 2.5. Histopathological Evaluation

At the end of the experiment, the mice were euthanized, and serum, spleen, and colon tissue were collected. The spleen wet weight was measured, then the spleen index was calculated as the spleen wet weight (mg) divided by the body weight (g). The entire colon was flushed with ice-cold PBS, then placed neatly on gauze, and the length of the colon was measured and recorded. Colon tissue samples from the middle region of the colon were fixed with 4% paraformaldehyde and embedded in paraffin. To assess inflammation, colon tissues were cut into sections in 4 *μ*m thickness and hematoxylin-eosin (HE) staining was performed. The extent of histopathological changes, including inflammatory cells infiltration (0–3), crypt injury (0–4), Epithelia damage (0–3), and presence or absence of edema (0 or 1), was graded according to previous studies (Table 2) [40].

### 2.6. AB-PAS Staining

The colon mucus secretion capacity of mice in each group was analyzed by Alcian blue periodic acid Schiff (AB-PAS) staining. After dewaxing and hydration, the paraffin sections of the colon tissues of mice were stained with allicin blue staining solution for 15 mins and rinsed with tap water until colorless. Secondly, stain with 0.5% periodic acid solution for 15 mins, rinse with tap water, and rinse twice with distilled water. Thirdly, the tissues were soaked in Schiff reagent at room temperature for 30 mins in the dark and rinsed for 5 mins. Then the tissue was dehydrated with gradient ethanol (75%, 95%, and anhydrous ethanol, respectively) for 5 mins, and xylene transparent for 5 mins. Finally, neutral gum was sealed, and the results were observed under an optical microscope after drying.

### 2.7. Cytokines and MPO Activity Determination by ELISA

Serum was extracted from blood after centrifugation at 850 ×g for 20 min, 4°C. IL-1*β* levels were measured using commercial ELISA kits (Shanghai Enzyme-Linked Biotechnology Co., Ltd.), following the kit instructions. The colon tissue was weighed and homogenized in cold normal saline and then centrifuged at 10,000*g* for 10 mins at 4°C. The supernatant was collected and then tested by ELISA kits according to the manufacturer's instructions to detect the levels of MPO, TNF-*α*, IL-10, and TGF-*β* (Shanghai Enzyme-Linked Biotechnology Co., Ltd.) in the supernatant of the homogenate. BCA protein assay kit (Tiangen Biotech (Beijing) Co., Ltd.) was used to determine the protein concentration of the supernatant in the homogenate.

### 2.8. Western Blotting Analysis

Colon tissues were homogenized in RIPA buffer containing 1% PMSF and phosphatase inhibitors. Supernatants were collected, and protein concentration was determined with a BCA protein assay kit (Solarbio Life Science, Beijing, China). Equal amounts of protein were loaded onto 8–10% sodium dodecyl sulfate-polyacrylamide gel electrophoresis (SDS-PAGE) and transferred onto polyvinylidene fluoride (PVDF) membranes (Bio-Rad Laboratories, Inc). After blocking with 5% skim milk or 5% bovine serum albumin (BSA) in TBST at room temperature for 2 h, membranes were incubated at 4°C overnight with primary antibodies against Occludin (1 : 800) and ZO-1 (1 : 1,000), TLR4 (1 : 1,000), MyD88 (1 : 1000), NF-*κ*B (p65) (1 : 1,000), p-NF-*κ*B (p-p65) (1 : 1,000), COX-2 (1 : 1,000), and GAPDH (1 : 10000). On the next day, the membranes were washed with TBST three times, followed by incubation with horseradish peroxidase (HRP)-conjugated goat anti-rabbit IgG antibody for 40 min at room temperature. Finally, the membranes were exposed using the ECL system (Tanon, China). Image J software was used for quantitative analysis (Table 3).

### 2.9. Statistics Analysis

The data were expressed as mean ± standard deviation (‾*X* ± SD) and all statistical differences were analyzed by the SPSS computer program (version 26.0). Multiple comparisons were performed using one-way analysis of variance (ANOVA) followed by Dunnett's multiple comparisons test. A value of *p* < 0.05 or *p* < 0.01 was considered as statistical significance. The GraphPad Prism software (version 8.02) is used for the visual display of all results.

## 3. Results

### 3.1. Jat Attenuated Symptoms of the DSS-Induced Colitis Mice

**Figure 1:**
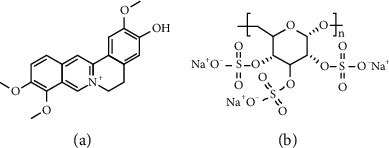
(a) The chemical structure of Jatrorrhizine. (b) The chemical structure of dextran sulfate sodium (DSS).

**Figure 2:**
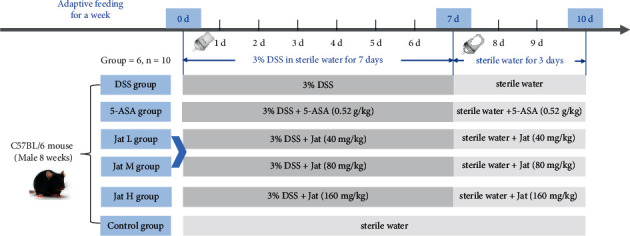
Experimental design of the induction of DSS colitis and drug treatment. (After one week of acclimatization, the mice were randomly divided into the control group, DSS group, 5-ASA group, and Jat groups. Except for the control group, other groups were given 3% DSS in sterile water to establish UC animal model. 5-ASA group and the Jat L, M, and H groups were administered orally once a day for 10 days at the same time as modeling. 7 days later, the water containing 3% DSS was replaced with sterile water, and each administration group continued to be administered for 3 days, and the mice were euthanized on the last day of the experiment).

**Figure 3:**
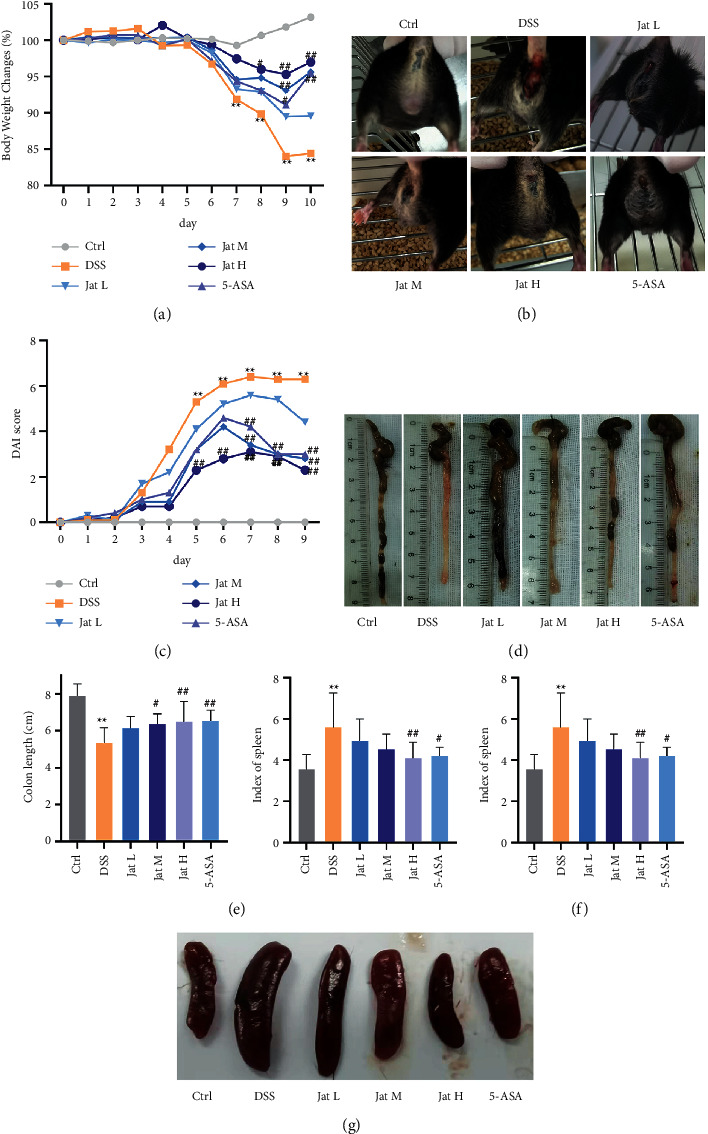
Jatrorrhizine attenuates DSS-induced UC in mice. (a) Body weight change in each group; (b) Blood in the stool of mice in different groups; (c) DAI score in each group; (d, e) Colon length in each group; (f, g) Spleen index and macroscopic appearances of the spleen. All data were presented as mean ± SD. (Ctrl: control group; DSS: DSS group; 5-ASA: mesalazine group; Jat L: Jatrorrhizine 40 mg/kg dose group; Jat M: Jatrorrhizine 80 mg/kg dose group; Jat H: Jatrorrhizine 160 mg/kg dose group; ^*∗*^*p* < 0.05 and ^*∗∗*^*p* < 0.01 vs. Control group; ^##^*p* < 0.05 and ^##^*p* < 0.01 vs. DSS group).

The structure of Jat and DSS are shown in Figures 1(a) and 1(b). In order to explore the therapeutic and preventive effect of Jat on UC, we established the acute colitis model by administering DSS, of which the experimental treatment schedule is shown in Figure 2. As shown in Figure 3(a), compared with the control group, the weight of the mice in the DSS group decreased significantly. While the weight loss of the mice in the Jat H group and 5-ASA group was significantly improved compared with the DSS group, the weight loss of the Jat M group was also improved, but not as obvious as in the Jat H group. DAI was the comprehensive index for assessing UC development. Mice treated with Jat M, H group or 5-ASA ameliorate the severity of colitis which is demonstrated by DAI scores (Figures 3(b) and 3(c)), compared to the DSS group. Moreover, the colon length of the DSS group was significantly shorter compared with the control group, while mice in the Jat treatment group showed a dose-dependent increase in colon length (*p* < 0.05) (Figures 3(d) and 3(e)). However, no obvious colon length change was observed in the low-dose JAT group compared to the DSS group. In addition, the increased spleen index was related to the severity of colitis. Both Jat H and 5-ASA groups significantly suppressed the DSS-mediated increase in the spleen index, shown as the ratio of spleen wet weight to body weight (*p* < 0.05, Figures 3(f) and 3(g)).

### 3.2. Jat Suppressed Colon Tissue Injury in DSS- Induced Colitis Mice

**Figure 4:**
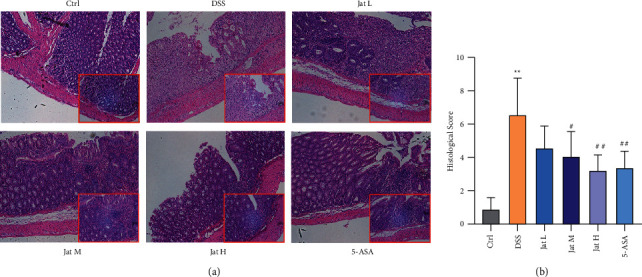
The effect of Jatrorrhizine on histopathological damage of colon in mice with UC (100×). (a) Macroscopic appearances of the colon; (b) Histological score in different groups. All data were presented as mean ± SD. (Ctrl: control group; DSS: DSS group; 5-ASA: mesalazine group; Jat L: Jatrorrhizine 40 mg/kg dose group; Jat M: Jatrorrhizine 80 mg/kg dose group; Jat H: Jatrorrhizine 160 mg/kg dose group; ^*∗*^*p* < 0.05 and ^*∗∗*^*p* < 0.01 vs. Control group; ^#^*p* < 0.05 and ^##^*p* < 0.01 vs. DSS group).

The histopathological analysis of the colons of mice with DSS-induced colitis was further assessed using HE staining. As shown in Figure 4(a), diffuse infiltration of inflammatory cells, mucosal erosion, congestion, edema, and crypt damage in colonic tissue were observed in the DSS group, while such changes were significantly suppressed in the Jat high and middle dose groups or 5-ASA-treated groups. Furthermore, the microscopic scores in Jat M and Jat H treated or 5-ASA treated mice were significantly reduced when compared to that of the DSS group (*p* < 0.01) (Figure 4(b)). These results indicated that Jat could attenuate the gross symptoms of DSS-induced colitis in mice and ameliorate colonic injury.

### 3.3. Jat Enhanced the Secretion of Colonic Mucin in DSS-Induced Colitis Mice

**Figure 5:**
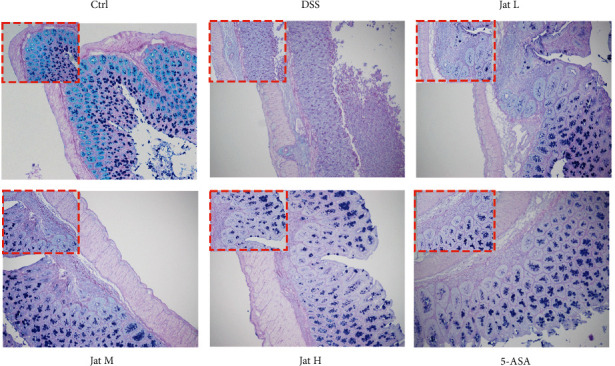
AB-PAS staining results of the therapeutic effect of Jatrorrhizine on UC mice. (AB-PAS staining, 100×). (Ctrl: control group; DSS: DSS group; 5-ASA: mesalazine group; Jat L: Jatrorrhizine 40 mg/kg dose group; Jat M: Jatrorrhizine 80 mg/kg dose group; Jat H: Jatrorrhizine 160 mg/kg dose group).

The loss of mucin-producing cells is one of the characteristics of colitis. Observing the change in goblet cell number and mucopolysaccharide content is an important morphological basis for the diagnosis of intestinal function and structural changes [41, 42]. In AB-PAS staining, the acidic mucous substances were dyed lake blue by AB staining, and the neutral mucous substances were dyed purple blue by PAS staining. As shown in Figure 5, AB-PAS staining showed that stained positive goblet cells of colonic mucosa were densely distributed on both sides of the crypt with regular and full shape in a normal group of mice. Meanwhile, in the DSS group, the mucosal layer was exfoliated, few crypts remained in the lamina, and the proportion of positive staining goblet cells containing mucus decreased obviously. However, the expression and distribution of mucin in goblet cells were significantly increased after treatment with Jat and 5-ASA, and the expression and distribution of mucin in goblet cells in the Jat high dose treatment group was the most obvious increase compared with the DSS group.

### 3.4. Jat Alleviated Inflammatory Response in DSS-Induced Colitis Mice

**Figure 6:**
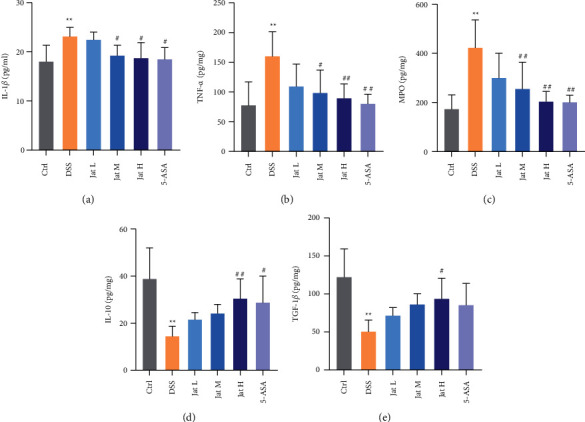
The effect of Jatrorrhizine on inflammatory cytokines levels in mice with UC.; (a) The levels of IL-1*β* in serum; (b) The levels of TNF-*α* in colon tissue; (c) The levels of MPO in colon tissue. (d) The levels of IL-10 in colon tissue; (e) The levels of TGF-*β* in colon tissue. All data were presented as mean ± SD. (Ctrl: control group; DSS: model group; 5-ASA: mesalazine group; Jat L: Jatrorrhizine 40 mg/kg dose group; Jat M: Jatrorrhizine 80 mg/kg dose group; Jat H: Jatrorrhizine 160 mg/kg dose group; ^*∗*^*p* < 0.05 and ^*∗∗*^*p* < 0.01 vs. Control group; ^#^*p* < 0.05 and ^##^*p* < 0.01 vs. DSS group).

As imbalance of pro/anti-inflammatory factors plays a critical role in the pathogenesis of UC [43, 44], an ELISA assay was performed. The results showed that the level of IL-1*β* in the serum was significantly increased in the DSS group compared to the control group, which was remarkably inhibited by Jat M, Jat H, and 5-ASA groups (Figures 6(a)). Additionally, we also measured the levels of TNF-*α*, and MPO in the colon tissues. The results showed that the levels of TNF-*α*, and MPO were significantly suppressed upon Jat treatments with high, medium dosage, and 5-ASA as compared to the DSS group (Figures 6(b) and 6(c)). Moreover, the levels of IL-10, and TGF-*β* were measured to assess anti-inflammatory response in the colon tissues. As shown in Figures 6(d) and 6(e), treatment with Jat H could upregulate the levels of IL-10, and TGF-*β* in the colon tissue after administrating with DSS.

### 3.5. Jat Improved Intestinal Barrier Function in DSS-Induced Colitis Mice

**Figure 7:**
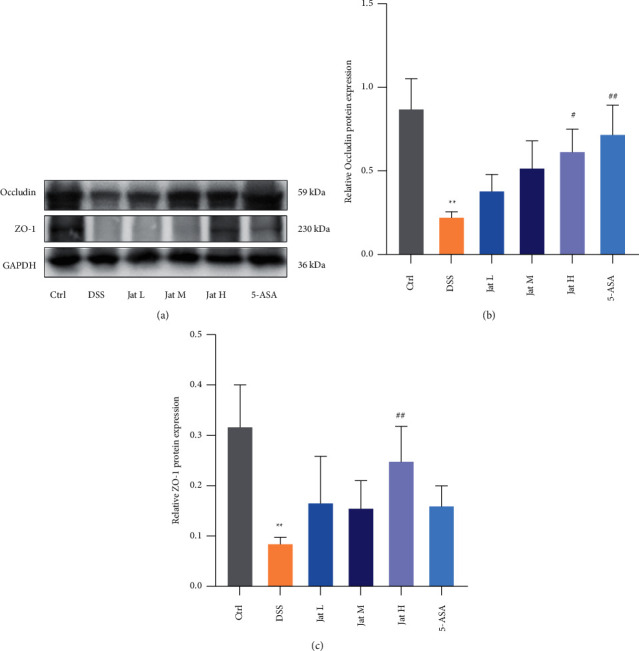
Effect of Jatrorrhizine on the protein expressions of Occludin and ZO-1 in DSS-induced UC in mice. (a) Western blotting images of Occludin, ZO-1 and GAPDH. (b) Relative occludin protein expression in colon tissue. (c) Relative ZO-1 protein expression in colon tissue. All data were presented as mean ± SD. (Ctrl: control group; DSS: DSS group; 5-ASA: mesalazine group; Jat L: Jatrorrhizine 40 mg/kg dose group; Jat M: Jatrorrhizine 80 mg/kg dose group; Jat H: Jatrorrhizine 160 mg/kg dose group; ^*∗*^*p* < 0.05 and ^*∗∗*^*p* < 0.01 vs. Control group; ^#^*p* < 0.05 and ^##^*p* < 0.01 vs. DSS group).

TJ is important constituent of the intestinal mucosal barrier, which is critical in protecting against inflammation. As shown in Figures 7(a)–7(c), the protein expressions of Occludin and ZO-1 in the DSS-treated group were decreased compared with those of the control mice, which indicated the integrity of the mucosal barrier was disrupted. However, treatment using Jat and 5-ASA helped protect the intestinal barrier by promoting the expressions of Occludin and ZO-1 in the DSS-induced mice (Figures 7(a)–7(c)).

### 3.6. Jat Suppressed TLR4/MyD88/NF-*κ*B Signaling Pathway in DSS-Induced Colitis Mice

**Figure 8:**
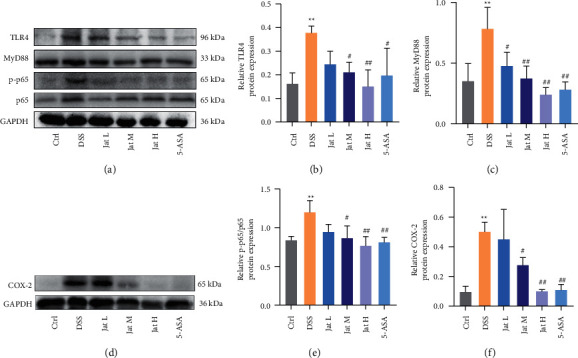
Effect of Jatrorrhizine on the protein expressions of TLR4, MyD88, p-p65, and COX-2 in DSS-induced UC in mice. (a) Western blotting images of TLR4, MyD88, p-p65, p65, and GAPDH. (b) Relative TLR4 protein expression in colon tissue. (c) Relative MyD88 protein expression in colon tissue. (d) Western blotting images of COX-2 and GAPDH. (e) Relative p-p65/p65 protein expression in colon tissue. (f) Relative COX-2 protein expression in colon tissue. All data were presented as mean ± SD. (Ctrl: control group; DSS: DSS group; 5-ASA: mesalazine group; Jat L: Jatrorrhizine 40 mg/kg dose group; Jat M: Jatrorrhizine 80 mg/kg dose group; Jat H Jatrorrhizine 160 mg/kg dose group; ^*∗*^*p* < 0.05 and ^*∗∗*^*p* < 0.01 vs. control group; ^#^*p* < 0.05 and ^##^*p* < 0.01 vs. DSS group).

To explore the anti-inflammatory signaling pathway, we further evaluated whether Jat could exert a central anti-inflammatory effect associated with TLR4/MyD88/NF-*κ*B signaling pathway. The results showed that compared with the control group, the expression of TLR4, MyD88, and the level of p-p65 was significantly upregulated after DSS administration (Figure 8(a)). However, Jat treatments with high or medium dosage could markedly decrease the protein levels of TLR4, MyD88, and p-p65 (Figures 8(a)–8(c), and 8(e)). As shown in Figures 8(a) and 8(e), Jat could significantly decrease the p-p65/p65 ratio in DSS-induced mice, indicating the inhibition of NF-*κ*B activity (Figures 8(a) and 8(e)). In addition, we also explored the expression of COX-2 in colon tissues. The results showed that the protein expression of COX-2 was increased in the DSS group compared with the control group. On the contrary, Jat M, Jat H, and 5-ASA treatments significantly reduced the expressions of COX-2 in the DSS administrated mice group (Figures 8(d) and 8(f)).

## 4. Discussion

Clinical UC patients typically have abdominal pain, rectal bleeding, and diarrhea [2]. The widely used mouse colitis model induced by DSS is characterized by persistent body weight loss, diarrhea, and rectal bleeding, which can be used to calculate DAI scores [8, 37]. Therefore, in this study, we established the experimental mice model induced by DSS to evaluate the therapeutic effect of Jat against UC. It is reported that intestinal inflammation is usually associated with diarrhea and decreased food intake. Therefore, the body weight is significant in estimating colitis severity [8]. As the previous study described, DSS-induced colitis mice may gain a little weight in the first three days and gradually lose weight once the bleeding starts [37]. In our investigation, a small weight gain was observed in the mice induced by DSS for the first three days. Whereas the body weight of the mice rapidly declined with the initiation of bleeding, which is associated with DAI scores. As shown in Figure 3, Jat therapy could significantly attenuate the colonic inflammatory symptoms caused by DSS, such as body weight loss, fecal occult blood, and DAI scores. Additionally, the shortened colon length and the increased spleen index were observed in the DSS group, which were generally correlated with the degree of inflammation [36]. Nevertheless, treatment with Jat remarkably extended the colon length and decreased the spleen index in DSS-induced colitis mice. These findings indicated that Jat ameliorated the symptoms of colitis. For further exploration, histopathology of the colonic tissues was studied. Observation of HE staining showed that Jat could significantly reduce the colonic tissue damage and lead to a lower histological score as compared to the DSS group, which was manifested in improving DSS-induced erosion of surface epithelial cells and destruction of the crypt accompanied by inflammatory cells infiltrating. These results preliminarily confirmed the protective and therapeutic effect of Jat against UC.

The imbalance of pro/anti-inflammatory factors is also key components in the pathogenesis of UC. Thus, drugs that could regulate the pro/anti-inflammatory mediators may afford an effective strategy for the therapy of UC [43, 44]. Therefore, we performed ELISA tests to assess the inflammatory level. The results showed that Jat could effectively reduce the expression levels of inflammatory mediators compared with the DSS group, such as IL-1*β*, TNF-*α*, and MPO. Besides that, treatment with Jat could also upregulate the levels of IL-10, and TGF-*β* in the colon tissue compared with the DSS group. Recent studies reported that DSS colitis severity is associated with increased production of various inflammatory mediators such as TNF-*α*, IL-1*β*, and MPO activity; and IL-10 and TGF-*β* drive anti-inflammatory response to prevent colitis [44, 45]. Taken together, these data further demonstrated that Jat could regulate the expression of these inflammatory mediators, which indicated Jat might provide an effective treatment for the therapy of UC. Furthermore, COX-2 is critical in the inflammation of chemical induced mice and 5-ASA is known to be an inhibitor of COX-2 [46]. Therefore we also explored the protein expression of COX-2 in colon tissues. The results showed that 5-ASA, medium or high doses of Jat treatments could significantly reduce COX-2 expression in DSS-treated mice.

The ability of the intestine to adequately retain luminal bacteria and chemicals while maintaining the capability to absorb nutrients is known as intestinal mucosal barrier function. The intestinal mucosal barrier includes mucin proteins mainly produced by goblet cells on the surface of the intestinal lumen, intestinal epithelial cells, and immune cells in the lamina propria [13]. Accumulating evidence has indicated that intestinal mucosal barrier disruption is critical for the development and progression of UC [5, 11, 13]. The degree of mucosal protection is largely regulated by the level of mucin glycosylation. When pathogens are present, mucin overexpression facilitates both the maintenance of the mucous protective layer and the downstream flushing of the harmful bacteria. Once the mucus layer is destroyed, the pathogens can access the underlying epithelium [47]. AB-PAS staining is frequently employed as a means of comprehensively detecting mucin since it may disclose acidic compounds as well as neutral mucinous proteins and glycogen [48]. According to the AB-PAS results, administration of DSS caused the intestinal mucosal structure disorder in mice with colitis, and the number of goblet cells and mucin levels was dramatically reduced. In contrast, the number of goblet cells and cell counts in the colon tissues of mice given Jat were significantly higher than those in the DSS group. Moreover, TJ localized at the interface between epithelial cells is crucial for maintaining the integrity of intestinal epithelial cells [49]. ZO-1 and Occludin are the main proteins comprising TJ, which prevent antigens and microorganisms in the intestinal lumen across the epithelium [5]. It has been reported that expressions of TJ proteins were changed in patients with UC and the impaired integrity of intestinal epithelial TJ proteins is a key component in the pathogenesis of UC [14, 15]. Protecting the integrity of the intestinal barrier may be the main target for the treatment of UC [50]. Therefore, in this study, we investigated the expression of ZO-1, Occludin in colon tissue. Our results showed that Jat, especially Jat high dosage group can significantly restore the protein expression of ZO-1, Occludin after DSS treatment, demonstrating the protective effect of Jat on the integrity of the epithelial barrier. These results together suggested that the protective effect of Jat on DSS-induced colitis may be closely related to the enhancement of mucin synthesis and improvement of epithelial TJ.

TLR4, a member of the Toll-like receptor family of proteins, is a type of pathogen pattern recognition receptor (PRR) that plays a central role in the gut innate immune defense [51]. Activation of TLR4 initiates the activation of MyD88, an important downstream regulator essential for TLR signaling, which triggers a signal transduction cascade leading to induction and activation of NF-*κ*B [52, 53]. These results cause translocation of NF-*κ*B p65 from the cytoplasm to the nucleus, and then NF-*κ*B p65 binds to DNA, facilitating the expression of proinflammatory mediators including COX-2 and production of proinflammatory cytokines such as IL-1*β* and TNF-*α*, thereby resulting in intestinal injury [20, 54–56]. Evidence indicated that TLR4 is highly upregulated in Inflammatory Bowel Disease (IBD) and DSS-induced UC in mice [18, 19]. Recent studies have shown that the TLR4/MyD88/NF-*κ*B signal-transducing pathway regulates the inflammatory response and plays a critical role in the progress of UC [16]. Therefore, inhibiting the TLR4/MyD88/NF-*κ*B signaling pathway is an attractive approach for the therapy of colitis. In this study, the results showed that TLR4, MyD88, and p-p65 expressions were significantly increased in the treatment of the DSS group. Moreover, the high dose group of Jat showed better results than other groups. These results suggest that Jat may exert the anti-inflammatory effect by inhibiting the activation of the TLR4/MyD88/NF-*κ*B pathway. Interestingly, an early study found that the connection between the mucosal inflammatory response and the disruption of epithelial barrier function depends heavily on NF-*κ*B [20]. A study's findings indicated that the expression of COX-2 in the intestinal epithelial cell is upregulated in a TLR4 and MyD88-dependent fashion [57]. Given this background, our findings suggest that an internal link may exist between the suppression of inflammatory mediators, blocking of the TLR4/MyD88/NF-*κ*B pathway, and restoration of intestinal barrier function in Jat ameliorate DSS-induced colitis.

## 5. Conclusion

In conclusion, it is the first time that Jat has been demonstrated to be a potential agent for UC treatment with a notable therapeutic effect on DSS-induced UC. The mechanism of Jat against UC may be related to suppressing TLR4/MyD88/NF-*κ*B signaling pathway and regulating the intestinal barrier function. However, the detailed mechanisms of Jat at the cellular and molecular levels, as well as in other animal models of UC, remain to be thoroughly investigated.

## Figures and Tables

**Table 1:** Criteria for disease activity index (DAI).

Score	Weight loss (%)	Stool consistency	Occult blood
0	None	Normal	Negative
1	1–5	Soft but still formed	Weakly positive
2	6–10	Soft	Positive
3	11–20	Very soft and wet	Blood traces in stool visible

**Table 2:** Histological colitis score.

Inflammatory cells infiltration	Crypt injury	Epithelia damage	Score
None	Normal	None	0
Mild	Basal1/3damage	Mucosa	1
Moderate	Basal 2/3damage	Submucosa	2
Severe	Crypt lost, surface epithelium present	Transmural	3
Extremely severe	Crypt and surface epithelium lost		4

**Table 3:** Antibodies information.

Antibodies	Dilution	Manufacturers	Cat.no.
Phospho-NF-*κ*B p65 Rabbit mAb	1 : 1000	Cell Signaling Technology	3033
NF-*κ*B p65 Rabbit mAb	1 : 1000	Cell Signaling Technology	8242
TLR4	1 : 1000	Proteintech	19811-1-AP
Zonula occludens-1 (ZO-1)	1 : 1000	Proteintech	21773-1-AP
Learn more about occludin from ScienceDirect's “AI-generated Topic Pages”>Occludin	1 : 800	Proteintech	27260-1-AP
GAPDH	1 : 10000	Proteintech	10494-1-AP
MyD88	1 : 1000	Affinity Biosciences	AF5195
COX-2	1 : 1000	BIOSS	bs-0732R
Goat anti-Rabbit IgG (H&L)	1 : 10000	ZENBIO	511203

## Data Availability

The data used to support the findings of this study are available from the corresponding author upon reasonable request.
